# TGFβ signaling plays a critical role in promoting alternative macrophage activation

**DOI:** 10.1186/1471-2172-13-31

**Published:** 2012-06-15

**Authors:** Dapeng Gong, Wei Shi, Sun-ju Yi, Hui Chen, John Groffen, Nora Heisterkamp

**Affiliations:** 1 Division of Hematology/Oncology, Ms#54, Children’s Hospital Los Angeles, 4650 Sunset Boulevard, Los Angeles, CA, 90027, USA; 2 Developmental Biology and Regenerative Medicine Program, Saban Research Institute, Children's Hospital Los Angeles, Los Angeles, CA, USA

**Keywords:** TGFβ, Macrophage polarization, Lung, Alveolar macrophage, M1, M2, Hematopoietic, Inflammation, TGFBR2, LGALS3

## Abstract

**Background:**

Upon stimulation with different cytokines, macrophages can undergo classical or alternative activation to become M1 or M2 macrophages. Alternatively activated (or M2) macrophages are defined by their expression of specific gene products and play an important role in containing inflammation, removing apoptotic cells and repairing tissue damage. Whereas it is well-established that IL-4 can drive alternative activation, if lack of TGFβ signaling at physiological levels affects M2 polarization has not been addressed.

**Results:**

*Vav1-Cre x TβRII*^*fx/fx*^ mice, lacking TβRII function in hematopoietic cells, exhibited uncontrolled pulmonary inflammation and developed a lethal autoimmune syndrome at young age. This was accompanied by significantly increased numbers of splenic neutrophils and T cells as well as elevated hepatic macrophage infiltration and bone marrow monocyte counts. *TβRII*^*-/-*^ CD4^+^ and CD8^+^ T-cells in the lymph nodes and spleen expressed increased cell surface CD44, and CD69 was also higher on CD4^+^ lymph node T-cells. Loss of TβRII in bone marrow-derived macrophages (BMDMs) did not affect the ability of these cells to perform efferocytosis. However, these cells were defective in basal and IL-4-induced *arg1* mRNA and Arginase-1 protein production. Moreover, the transcription of genes that are typically upregulated in M2-polarized macrophages, such as *ym1, mcr2 and mgl2,* was also decreased in peritoneal macrophages and IL-4-stimulated *TβRII*^*-/-*^ BMDMs. We found that cell surface and mRNA expression of Galectin-3, which also regulates M2 macrophage polarization, was lower in *TβRII*^*-/-*^ BMDMs. Very interestingly, the impaired ability of these null mutant BMDMs to differentiate into IL-4 polarized macrophages was Stat6- and Smad3-independent, but correlated with reduced levels of phospho-Akt and β-catenin.

**Conclusions:**

Our results establish a novel biological role for TGFβ signaling in controlling expression of genes characteristic for alternatively activated macrophages. We speculate that lack of TβRII signaling reduces the anti-inflammatory M2 phenotype of macrophages because of reduced expression of these products. This would cause defects in the ability of the M2 macrophages to negatively regulate other immune cells such as T-cells in the lung, possibly explaining the systemic inflammation observed in *Vav1-Cre x TβRII*^*fx/fx*^ mice.

## Background

Transforming growth factor beta (TGFβ) is a multi-functional cytokine that regulates cell proliferation, differentiation, migration and survival. It plays a critical role in development, wound healing and immune responses through its regulatory effects on many cell types including epithelial and hematopoietic cells [1]. Three isoforms (TGFβ1, β2 and β3), which are encoded by different genes and share high homology, have been identified in mammals. TGF-β1 is the predominant isoform expressed in immune cells, but all three isoforms have similar properties *in vitro*. However, *in vivo*, they have both redundant and distinctive functions because of spatial and temporal expression during development [2-4]. TGFβ signals are transmitted via a cell surface receptor complex consisting of the TGFβ type I receptor (TβRI) and TGFβ type II receptor (TβRII). To initiate signal transduction, TGFβ binds to TβRII, which in turn recruits TβRI, leading to the formation of a tetrameric receptor complex. The constitutively active serine/threonine kinase of TβRII phosphorylates TβRI, which activates Smad2 and Smad3 via phosphorylation. Activated Smad2/3 binds Smad4. Subsequently, the Smad2/3/4 complex translocates to nucleus to regulate gene expression [5]. Besides the Smad-dependent pathways, TGFβ also activates the Erk [6], PI3K [7], p38 and JNK [8] pathways, each of which results in a unique pattern of gene expression and thus physiological function.

The importance of TGFβ signaling in the immune system is highlighted by the finding that mice lacking TGFβ1 develop a severe lethal wasting syndrome within 3 weeks of birth, associated with a mixed inflammatory cell infiltration and lesions in different organs including the heart and lungs [9,10]. Since TβRII is the primary receptor for TGFβ, Leveen et al. [11] generated a conditional ablation model for TGFβ signaling using *TβRII*^fx/fx^ x *Mx1-Cre* mice. Upon deletion of the TβRII in the bone marrow of adult mice, a lethal inflammatory disorder that is similar to the phenotype of the TGFβ1-null mutants was observed. Such lethal disorder was transferable through bone marrow transplantation, indicating that a deficiency of TGFβ signaling in cells of bone marrow origin is sufficient to cause a lethal inflammatory disease [11]. Further studies on TGFβ1-null mutants or mice with bone marrow-specific deletion of TβRII showed, that TGFβ signaling is critical for T-cell immunity [12-14], whereas possible functional defects of macrophages lacking TGFβ signaling were largely overlooked.

Macrophages are important immune cells that have diverse biological functions, which are, to a large extent, determined by their activation states. Exposure to LPS and IFNγ induces macrophages to undergo classical activation and differentiate into M1 macrophages. M1 macrophages are characterized by the production of pro-inflammatory cytokines (TNFα and IL-6) and the induction of iNOS (NOS2), which is essential for generating reactive oxygen species such as NO [15,16]. Because of their pro-inflammatory and cytotoxic activities, M1 macrophages play a critical role in elimination of pathogens and initiation of inflammation. In contrast, when macrophages are exposed to IL-4 (or IL-13), they undergo alternative activation and polarize into M2 macrophages, which are anti-inflammatory. M2 macrophages produce anti-inflammatory cytokines (IL-10) and have increased expression of Arginase-1, which competes with iNOS for a common limiting substrate L-arginine [17]. M2 macrophages are primarily involved in phagocytosis of apoptotic cells, resolution of inflammation, tissue repair, and wound healing [18-21].

Although TGFβ plays a critical role in T-cell immunity, surprisingly, few studies have evaluated the effect of TGFβ signaling on macrophages. To begin to answer this question, we generated mice lacking TβRII and thus TGFβ signaling in hematopoietic cells using *TβRII*^*fx/fx*^ and *Vav1-Cre* mice. Vav1-Cre has been shown to direct Cre-mediated recombination in cells of the hematopoietic lineage [22]. Interestingly, our studies reveal that macrophages lacking TβRII have defects in expression of a set of genes that form the hallmark of the M2 polarizing program, suggesting that TGFβ signaling is needed for the alternative activation of macrophages.

## Results

### Mice lacking TβRII in hematopoietic cells develop a lethal inflammatory and autoimmune syndrome

**Figure 1:**
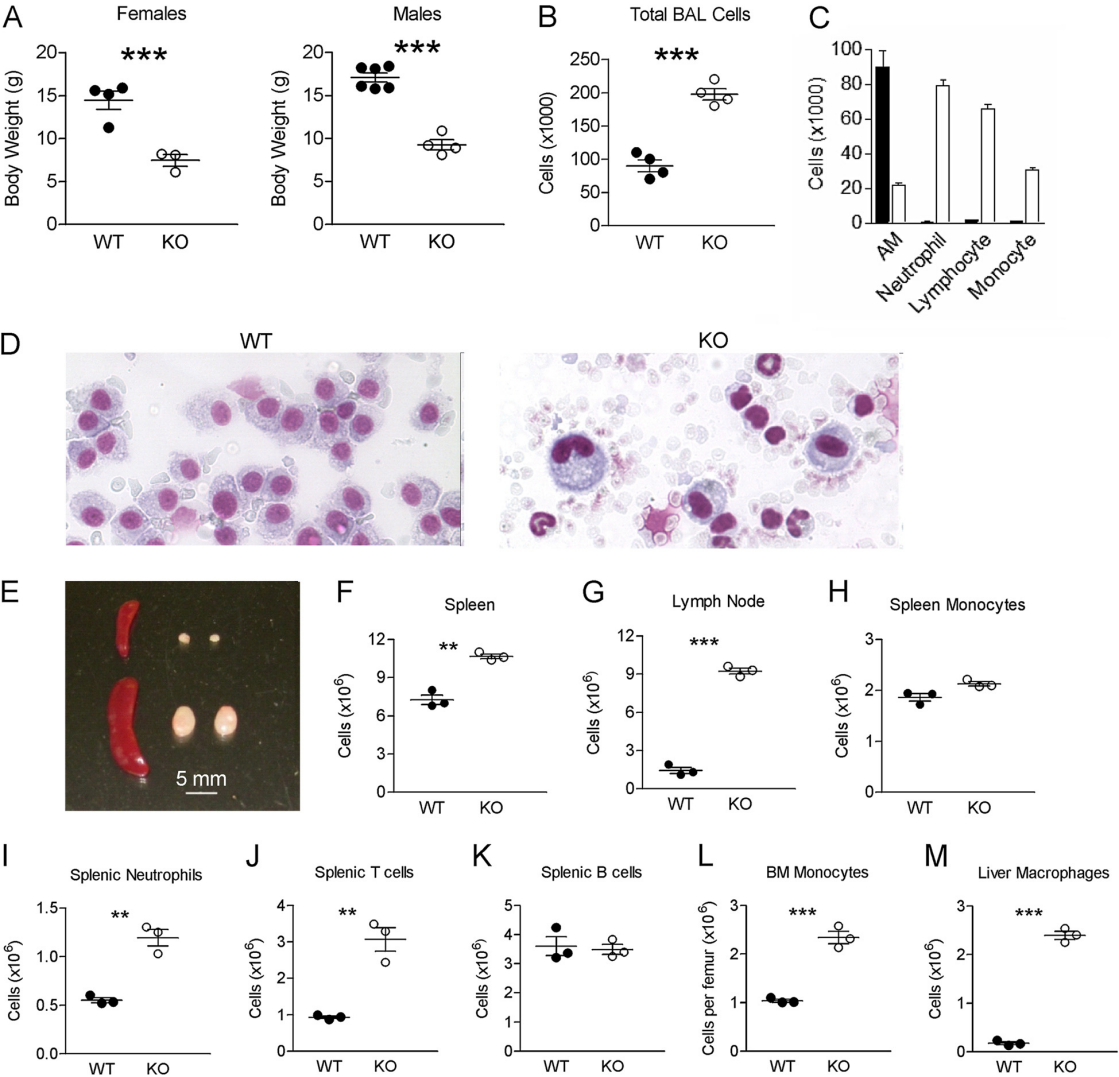
**Mice lacking TβRII in hematopoietic cells develop a lethal inflammatory and autoimmune syndrome.** (**A**) Body weight of male and female *Vav-Cre x TβRII*^*fx/fx*^*(TβRII*^*-/-*^) mice and littermate controls at postnatal day 18-21. (**B**) Total cell counts and (**C**) differential cell counts in BALF of *WT* and *TβRII*^*-/-*^ mice at postnatal day 18-21. Solid bar, *WT*; Open bar, *TβRII*^*-/-*^ samples. Differences are significant (p < 0.01) for all cell populations in panel C. (**D**) Wright-Giemsa-stained BALF cells from the indicated genotypes (18-day old littermates). Note the presence of a single cell type - the alveolar macrophage - in the control lung, whereas the *TβRII*^*-/-*^ sample contains neutrophils, monocytes and lymphocytes. (**E**) Photographic images of spleens and lymph nodes; total cell counts in (**F**) spleens and (**G**) lymph nodes; total numbers of (**H**) monocytes, (**I**) neutrophils, (**J**) T-cells and (**K**) B-cells in spleen; total counts of bone marrow monocytes (**L**) and liver macrophages (**M**) in *WT* (filled circles)and *TβRII*^*-/-*^ (KO, open circles) mice (n = 3, **p < 0.01; ***p < 0.001).

Vav1 is a hematopoietic-specific activator for Rac GTPases, and the *Vav1* promoter has been widely used to direct expression of genes selectively in the hematopoietic lineage [22]. Thus, in *Vav1-Cre x TβRII*^*fx/fx*^*(TβRII*^*-/-*^*)* mice, the *Vav1-Cre* transgene only ablates TβRII in hematopoietic cells. We found that mice lacking TβRII function in such cell types were ~50% lighter than wild type *(WT)* littermates (Figure 1A) and usually died at the age of 24-28 days. These *TβRII*^*-/-*^ mice showed classical hallmarks of acute lung inflammation. Total cell numbers in the BALF of *TβRII*^*-/-*^ mice were significantly higher than those of *WT* mice (Figure 1B). Whereas BALF cell populations of age-matched *WT* mice consisted, as expected, almost entirely of alveolar macrophages, those of *TβRII*^*-/-*^ mice instead contained many neutrophils, lymphocytes and monocytes (Figure 1 C and D).

Mice lacking TβRII in hematopoietic cells exhibited a normal-sized thymus but enlarged lymph nodes and spleen (Figure 1E), which correlated with a greatly increased cellularity of these tissues (Figure 1 F and G). *TβRII*^*-/-*^ spleens contained increased total neutrophil and T-cell numbers, while total monocyte and B-cell numbers were comparable between genotypes (Figure 1H-K). Strikingly, monocyte numbers in bone marrow, and macrophage infiltration in liver were significantly increased in *TβRII*^*-/-*^ mice (Figure 1 L and M). Since macrophage infiltration in liver is a sign of autoimmune disease, the increased infiltration of macrophages in the livers of *TβRII*^*-/-*^ mice is consistent with the observed inflammatory autoimmune syndrome. We next examined spleen, lymph nodes and thymus for the major T-cell subsets using FACS. Analysis of the thymus showed *WT* and *TβRII*^*-/-*^ mice had a similar distribution of CD4^+^, CD8^+^ and double positive (DP) or double negative (DN) subsets (Additional file 1: Figure S1 A and B). This indicates there were no abnormalities in T-cell development, which is consistent with a previous report that TGFβ signaling is not required for thymocyte differentiation [12]. We also found that CD25 expression on CD4^+^ T cells was comparable in thymus, spleen and lymph nodes of *WT* and *TβRII*^*-/-*^ mice (Additional file 1: Figure S1C), suggesting TβRII signaling does not regulate the development of T_reg_ cells and consistent with previous studies [23,24].

Analysis of the spleen and lymph nodes showed similar percentages of CD4^+^ and CD8^+^ T-cells, although their absolute numbers were increased in *TβRII*^*-/-*^ mice (not shown). Interestingly, however, there was a dramatic increase in expression of CD44 on T-cells. Around 20% of splenic CD4^+^ T-cells and 40% of CD8^+^ T-cells expressed CD44 in *WT* mice, while 90-95% of T-cells lacking TβRII had CD44 expression. This was also found in lymph nodes (Additional file 1: Figure S1 E and F). In addition, a significantly higher percentage of CD4^+^ T-cells in lymph nodes of *TβRII*^*-/-*^ mice expressed CD69 (Additional file: 1 Figure S1G). Since CD44 and CD69 are T cell activation markers, our data indicate that one physiological role of TβRII signaling is to suppress T cell activation.

### Expression of M2 markers on BMDMs is impaired in the absence of TβRII signaling

The lack of normal alveolar macrophages in the BALF of *TβRII*^*-/-*^ mice could be associated with intrinsic defects in those macrophages, or could be caused by the environment, since it is well-known that monocytes from the blood can differentiate into different types of macrophages depending upon environmental factors. Treatment with LPS and IFNγ will generate classically activated M1 macrophages, whereas treatment with IL-4 and dexamethasone generates M2a and M2c polarized macrophages, respectively. To examine macrophages lacking TβRII without the presence of other, possibly confounding, cell types, we isolated bone marrow-derived macrophage/monocytes (BMDMs) from the *TβRII*^*-/-*^ mice.

FACS analysis of *WT* and *TβRII*^*-/-*^ BMDMs after 11 days in culture with M-CSF, a macrophage growth factor, showed that cells of both genotypes were uniformly Ly6G^low^CD11b^hi^ and expressed F4/80, a pan-macrophage surface marker (Additional file 1: Figure S2A), indicating that the purity of the isolated BMDMs is very high (~99%). We then treated these BMDMs with stimuli that are typically used to generate different subsets of polarized macrophages. Additional file 1: Figure S2B illustrates that, as reported previously [15,20,25], these stimuli generate morphologically distinct populations. However, BMDMs with or without TβRII were morphologically similar. We also investigated the efficiency of Vav1-Cre-mediated ablation of the TGFβRII in these cells. Quantitative real-time PCR on DNA from BMDMs showed that, although the deletion was efficient, there was a residual signal of about 6% for TβRII present in Vav1-Cre x*TβRII*^fx/fx^ BMDMs (Additional file 1: Figure S2E).

We next assayed *WT* and *TβRII*^*-/-*^ BMDM for their ability to phagocytose apoptotic cells. As shown in Additional file 1: Figure S2C, there were no significant differences in the ability of M0 BMDMs with or without TβRII to perform efferocytosis. Next, we polarized them using different agents including LPS/IFNγ (M1), IL-4 (M2) and hTGFβ1. BMDMs polarized towards the M1 phenotype had significantly decreased ability to phagocytose dying cells (Additional file 1: Figure S2D), but no significant differences were observed between *WT* and *TβRII*^*-/-*^ BMDMs. We also tested migration. In contrast to bone-marrow derived neutrophils, which showed significant migration towards 0.1 ng/ml TGFβ1, bone marrow-derived macrophages did not migrate towards 5 ng/ml TGFβ1 (not shown).

**Figure 2:**
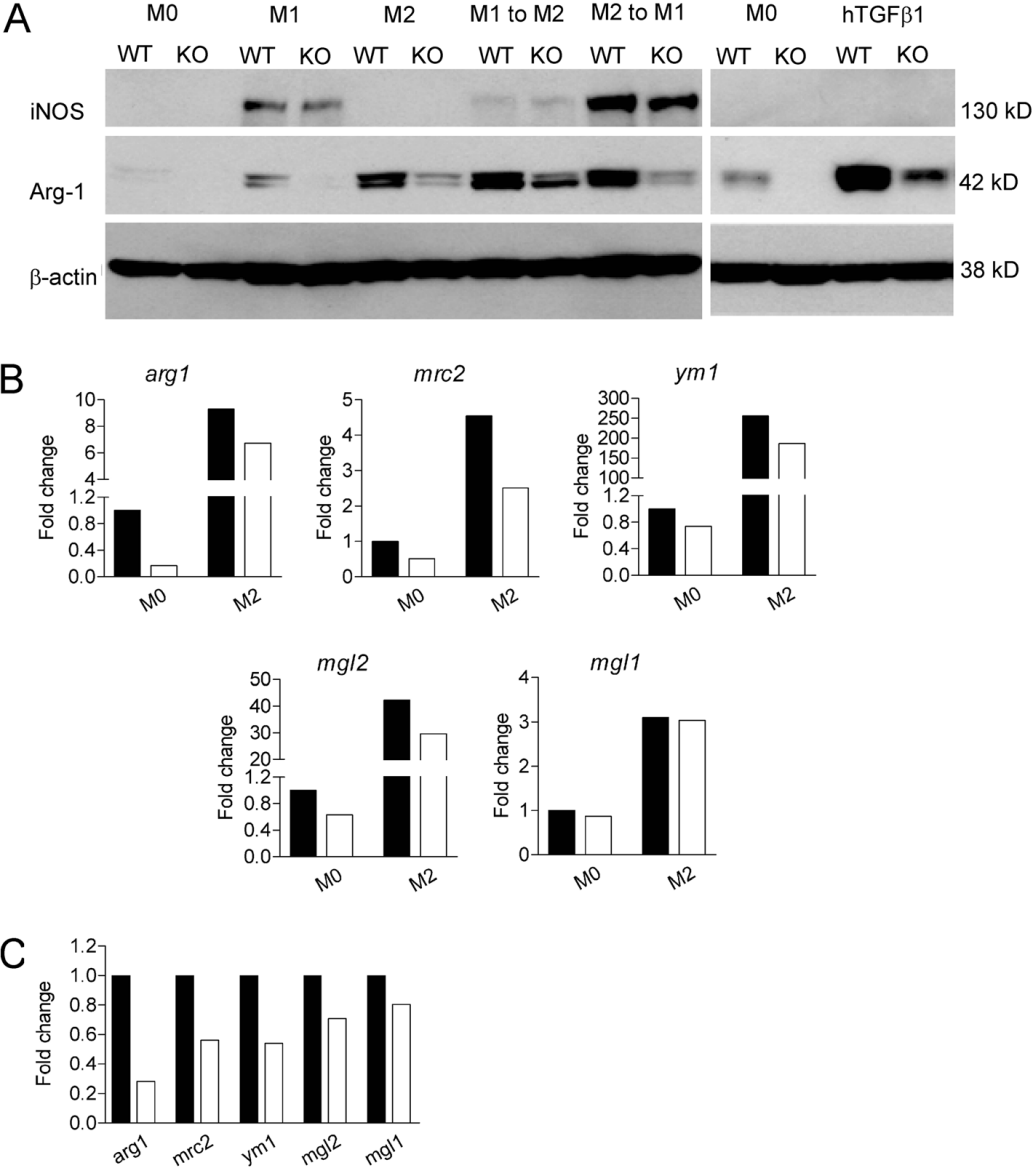
**Expression of M2 markers on BMDMs is impaired in the absence of TβRII signaling. (A**) Western blot analysis of *WT* and *TβRII*^*-/-*^ BMDMs cultured for 24 hrs with medium alone (M0), with LPS/IFNγ (M1), IL-4 (M2), or hTGFβ1. β-actin serves as a loading control. Increased Arg-1 in KO BMDM upon hTGFβ1stimulation due to residual WT cells, see Additional file 1: Figure S2E. (**B**) Real-time RT-PCR for the indicated genes after incubation for 24 hrs with medium alone (M0) or IL-4 (M2). (**C**) Real-time RT-PCR for the indicated genes in naïve peritoneal macrophages. Fold change is with respect to the expression level of M0 WT. Solid bar, *WT*; Open bar, *TβRII*^*-/-*^ peritoneal macrophages. The results shown are representative of one of two independently derived sets of BMDMs from different mice.

A classical readout for macrophage polarization states is the induction of either iNOS (M1) or Arginase-1 (M2), two enzymes that compete for the common substrate L-arginine. As illustrated in Figure 2A, iNOS was not expressed in M0 or M2 macrophages. Lack of TβRII allowed the normal production of iNOS upon stimulation with LPS/IFNγ. As reported previously [26], there was a baseline level of Arginase-1 (Arg-1) expression in non-polarized macrophages, which was enhanced by treatment with LPS. Remarkably, *TβRII*^*-/-*^ BMDMs had very low baseline levels of Arg-1, showed severely impaired induction of Arg-1 by LPS and had profoundly reduced induction of Arg-1 by IL-4 treatment. Similar defects in Arg-1 expression were obtained when M1-polarized (LPS/IFNγ-treated) *TβRII*^*-/-*^ macrophages were subsequently treated with IL-4 to re-polarize them to an M2 phenotype. Interestingly, macrophages lacking Smad3 had normal induction of Arg-1, indicating that the mechanism is Smad3-independent (Additional file 1: Figure S3).

TGFβ1 is generally regarded as an anti-inflammatory cytokine and it is frequently listed in the same category as IL-10 and glucocorticoids such as dexamethasone to generate M2c-polarized macrophages. However, we found that *WT* BMDMs reacted to hTGFβ1 stimulation with an increased expression of Arg-1 (Figure 2A), similar to the classical M2a stimulus IL-4, whereas dexamethasone (M2c) failed to induce Arg1 (not shown).

To examine if *TβRII* signaling only regulates Arg-1 expression, or that there are defects in the expression of other M2 markers, we next performed real-time RT-PCR for a number of molecules that are typically up-regulated in IL-4-polarized macrophages such as *ym1mrc2* and *mgl1/2*[27-30]. As shown in Figure 2B, lack of TβRII was associated with decreased basal and IL-4-induced transcription of *arg1* and correlated with decreased transcription of *mcr2, mgl2*, and *ym1*, which are also induced by IL-4. The transcription of *mgl1* was not affected. A similar pattern of reduced basal levels of M2 markers was also observed in naïve peritoneal macrophages (Figure 2C). Collectively, our data indicate that *TβRII* signaling is critical for the optimal expression of genes characteristic for M2 polarization.

### Lack of TβRII signaling is correlated with decreased galectin-3, β-catenin and phospho-Akt levels

The molecular pathways that underlie macrophage polarization are of intense interest due to the importance of this cell type in a wide array of pathologies, ranging from autoimmune disorders to cancer, and the desire to be able to modulate their polarization state. However, there are very few reports of null mutants in which macrophage polarization defects have been reported. One of these is Galectin-3 (*lgals3*). Macrophages lacking Galectin-3 show impaired IL-4-stimulated M2 polarization and *lgals3* null mutants have reduced TGFβ-associated liver fibrosis [31,32].

**Figure 3:**
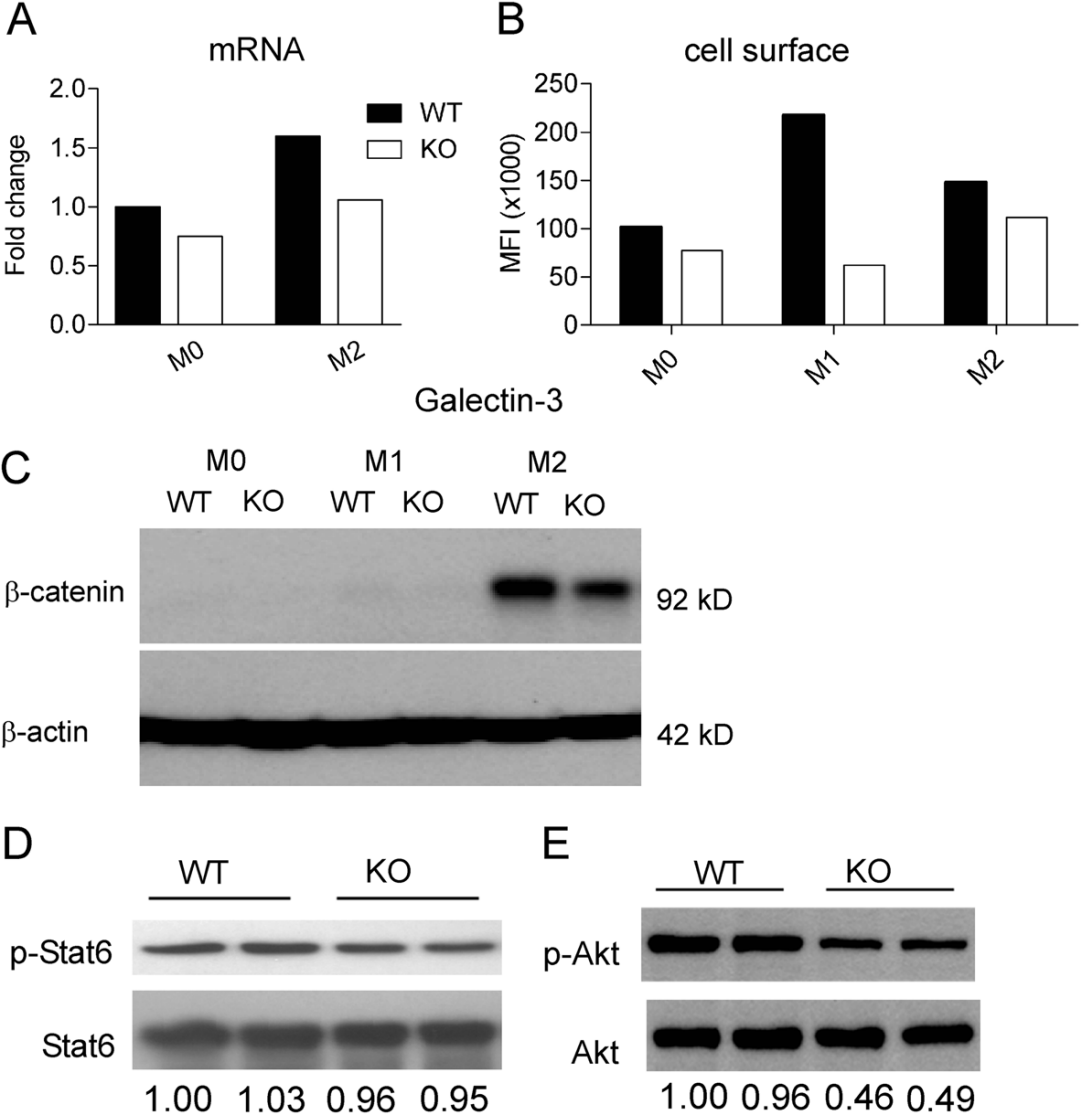
**Lack of*TβRII*signaling is correlated with decreased Galectin-3, β-catenin and phospho-Akt levels.** (**A**) Real-time RT-PCR for *lgals3* in BMDMs. Fold change is with respect to the expression of M0 WT. Solid bar, *WT*; Open bar, *TβRII*^*-/-*^*.* (**B**) Cell surface expression of Galectin-3 on *WT* and *TβRII*^*-/-*^ BMDMs measured using FACS. MFI, mean fluorescent intensity. The result shown is representative of one of two independently derived sets of BMDMs from different mice for both A and B. (**C**) Western blot analysis of β-catenin expression. BMDM were treated with medium alone (M0), LPS/IFNγ (M1), or IL-4 (M2). Results are representative of 3 pairs of matched *WT* and *TβRII*^*-/-*^ mice. Western blot analysis of phosphorylated-Stat6 (**D**) and phosphorylated-Akt (**E**) in WT and *TβRII*^*-/-*^ BMDMs that had been treated with IL-4 for 1 hr. Results are shown for 2 independent mice per genotype. The numbers at the bottom represent the band intensity ratio of phosphorylated protein versus total protein, normalized to lane 1 on each blot.

We therefore performed real-time RT-PCR for *lgals3*. Interestingly, *TβRII*^*-/-*^ BMDMs stimulated with IL-4 had reduced mRNA levels of *lgals3* (Figure 3A). To examine this in more detail, we stimulated *WT* and *TβRII*^*-/-*^ BMDMs with different polarizing agents and measured cell surface Galectin-3 expression using FACS. Whereas the expression of cell surface CD11b was comparable in these different treatment conditions (not shown), BMDMs lacking TβRII showed clearly decreased Galectin-3 expression, especially when exposed to LPS/IFNγ (Figure 3B).

The E-cadherin/β-catenin complex was recently shown to be a selective marker for alternatively activated macrophages [33]. Interestingly, when *WT* and *TβRII*^*-/-*^ BMDMs were stimulated by IL-4, β-catenin levels were ~35% lower in *TβRII*^*-/-*^ than in *WT* BMDMs (Figure 3C), further indicating that TβRII signaling promotes M2 macrophage polarization.

To explore the mechanism by which TGFβ contributes to IL-4-induced M2 polarization, we examined the downstream targets of IL-4. STAT6 phophorylation is a key signaling event downstream of the IL-4 receptor complex, and Akt also becomes activated in this pathway [34-36]. Thus we investigated if STAT6 and/or Akt are also involved in the IL-4 stimulated polarization that is co-regulated by TGFβ signaling. As shown in Figure 3D, p-Stat6 levels were comparable in *WT* and *TβRII*^*-/-*^ BMDMs stimulated with IL-4. However, p-Akt was decreased ~50% in the absence of TGFβ signaling (Figure 3E). This indicates that TGFβ contributes to IL-4-induced M2 polarization through co-signaling to Akt, which is one of the TGFβ1 non-Smad-associated signal transduction pathways reported in other cell types [37,38].

## Discussion

In the current study, we found that the phenotype of mice born without TβRII function in hematopoietic cells overlaps with that of mice born without systemic TGFβ1 production. In both cases, the null mutants develop a severe wasting syndrome at around the same time, at 3 weeks of age [9,10]. However, our mice exhibited splenomegaly and lymph node enlargement with a corresponding large increase in cellularity, whereas Shull et al. reported reduced size of spleen and Peyer’s patches [10].

We measured dramatically increased expression (15-40% in WT, 80-95% in KO) of the activation marker CD44, a receptor for the extracellular matrix (ECM) protein hyaluronic acid, on both CD4^+^ and CD8^+^ T cells in the spleen and lymph nodes. Similarly increased CD44 expression was also observed in mice transplanted with bone marrow of mice transgenic for a dominant negative TβRII construct [39]. CD69 is a C-type lectin that is expressed on the surface of all leukocytes during activation, and engagement of CD69 maintains high expression of membrane-bound TGFβ1 on T-cells [40]. We found that the numbers of CD4^+^ and CD8^+^ T-cells in the lymph nodes that expressed CD69 were around twice that of control cells. This result is consistent with the increased numbers of CD69^+^ T-cells found in the lymph nodes of mice transplanted with bone marrow from *TβRII*^fx/fx^ x *Mx1-Cre* mice and with the signs of T-cell activation in lymph nodes and spleen in those mice [12].

Alveolar macrophages are a specialized subclass of differentiated tissue macrophages that are generally regarded as regulatory and anti-inflammatory [41]. At three weeks of age, *Vav1-Cre x TβRII*^fx/fx^ mice developed spontaneous pulmonary inflammation in which few normal alveolar macrophages were found. The lack of normal alveolar macrophages could be explained by the pro-inflammatory environment in the lung, which would polarize peripheral blood monocytes that migrate into the lung to an M1 phenotype and/or by re-polarization of alveolar macrophages into proinflammatory M1 macrophages. Also, reduced production of monocytes in the bone marrow could result in lower levels of alveolar macrophages. However, we did not find increased levels of IFNγ, a classic proinflammatory cytokine, in the peripheral blood of the null mutant mice (Additional file 1: Figure S4) and there was no evidence for impaired generation of monocytes in the bone marrow of mice lacking hematopoietic TβRII.

To examine possible macrophage-intrinsic defects, we studied naïve, bone-marrow derived monocyte/macrophages for possible cell-intrinsic defects. This analysis showed an overall decreased ability of macrophages lacking TβRII signaling to produce the normal amounts of *arg1* mRNA and protein, either at basal levels or, more markedly, when BMDMs were polarized towards an M2 phenotype with IL-4. In agreement with the regulation of Arg-1 by TGFβ1, we found increased expression of Arg-1 in *WT* macrophages stimulated with this cytokine. Moreover, transcription of other M2 markers including *mgl2*, *ym1* and *mcr2*, but not *mgl1*, was also deceased in *TβRII*^-/-^ BMDMs, showing that signals through TβRII modulate the M2 transcription program.

Ym1 is a secretory lectin that promotes Th2 cytokine expression [42], whereas Mrc2 is a C-type receptor for mannose that promotes remodeling of the ECM and the uptake and degradation of collagen [29]. Both Mgl1 (clec10a) and Mgl2 as markers for alternatively activated macrophages are type C galactose-binding lectins with a different binding specificities to carbohydrate structures [43]. These results show that TGFβ signaling is responsible for the transcriptional upregulation of several M2-polarization genes in macrophages that are important for binding to carbohydrate structures present in the ECM and on other cells and are consistent with the role of TGFβ as a master regulator of ECM reorganization [44,45].

We also found that macrophages lacking TβRII produce less mRNA for *lgals3*, which encodes a β-galactoside-binding lectin, and have markedly reduced Galectin-3 cell surface expression on M1 (LPS/IFNγ-polarized) macrophages. Interestingly, Galectin-3 negatively regulates LPS-induced inflammation [46]. Moreover, BMDMs lacking *lgals3* have reduced Arg-1 production and are defective in M2 polarization [32]. These results suggest that some of the defects in *TβRII*^-/-^ BMDMs could be caused by the reduced Galectin-3 in those cells. Importantly, Akt phosphorylation was decreased in the IL-4-polarized M2 *TβRII*^-/-^ BMDMs. This suggests that TGFβ and IL-4 co-signal to maximally activate the Akt pathway. Although further experiments will be needed to determine the detailed molecular mechanisms through which TGFβ regulates M2 polarization, our findings suggest that modulation of the TGFβ signaling pathway may be a method to medically regulate macrophage polarization.

## Conclusions

Taken together, our results establish a critical biological role for TGFβ signaling in promoting the alternative activation of macrophages. Moreover, our results suggest a distinct contribution of monocytes/macrophages to the systemic inflammation observed in mice with defective TβRII function, because lack of TβRII signaling in macrophages inhibits the polarization of macrophages to an anti-inflammatory M2 phenotype, this could lead to defective down-regulatory interactions with other immune cells such as T-cells in the lung. These findings further advance our knowledge of the physiological role of TGFβ signaling and enhance our understanding of the regulation of macrophage activation states.

## Methods

### Animals

The *TβRII*^*fx/fx*^ mice [47] on a C57BL/6 background were obtained from the NCI Mouse Repository (Strain number: 01XN5) and were bred to *Vav1-Cre* mice, which were generously provided by Dr. Dimitris Kioussis (National Institute for Medical Research, UK) [22], to generate *Vav1-Cre x TβRII*^*fx/fx*^ mice and littermate controls. *TβRII*^*-/-*^ mice develop a wasting syndrome 24-28 days after birth. All animal studies were approved by the Institutional Animal Care and Use Committee (IACUC) of Children’s Hospital Los Angeles.

### Bronchoalveolar lavage

To prepare BALF, tracheas were exposed and cannulated with an 18-gauge angiocath. Lungs were lavaged five times with 0.8 ml of cold sterile PBS. Analysis of resident and recruited cells was performed on cells pooled from the five washes.

### Cytospin and differential staining

50-80,000 cells were spun onto glass slides at 800 rpm for 5 min in a Shandon Cytospin II Cytocentrifuge. Slides were stained using a Kwik-Diff stain kit (Thermo Fisher Scientific). Cells were differentially (morphologically) counted using light microscopy.

### Tissue culture and isolation of bone marrow-derived macrophages and peritoneal macrophages

Tissue culture reagents were from Invitrogen. LPS (cat no. L2630) was from Sigma-Aldrich. IL-4, hTGFβ1, IL-10 and IFNγ were purchased from PeproTech. The L929 cell line was from the American Type Culture Collection. The supernatant collected from L929 cultures that had grown for 7 days was used to prepare L929 conditioned media, as a source of M-CSF, for macrophage maturation and expansion. Bone marrow-derived macrophages (BMDMs) were prepared by maturing bone marrow cells, which were isolated from femur and tibiae of mice, in DMEM containing 15% FBS and 20% L929 conditioned media for 11 days. BMDMs were expanded and treated with IFNγ (10 ng/ml) and LPS (100 ng/ml) for M1 polarization and IL-4 (10 ng/ml) for M2 polarization. BMDMs were also treated with hTGFβ1 at 5 ng/ml or dexamethasone at 40 ng/ml. Peritoneal cells were harvested from the peritoneal cavity of mice by lavage. Cells from 3-4 mice of the same genotype were pooled and stained for FITC-Ly6G, PE-CD11b, PerCP-CD45 and APC-F4/80. Peritoneal macrophages (CD45^+^CD11b^+^Ly6G^-^F4/80^+^) were sorted out using a BD FACSAria Sorter. Sorted cells were lyzed in Trizol reagent (Invitrogen) for total RNA extraction.

### Real-time RT-PCR

Total RNA was prepared using RNeasy kits (Qiagen). cDNA was synthesized using a First Strand cDNA Synthesis kit (Invitrogen). The cDNA was amplified by 40 two-step cycles (15 sec at 95°C for denaturation of the DNA, 1 min at 60°C for primer annealing and extension). cDNA was analyzed using a SYBR green-based quantitative fluorescence method (Applied Biosystems) in duplicate. The PCR primers were as follows:

*GAPDH*: forward 5'-AGA GGG AAA TCG TGC GTG AC-3', reverse 5'-CAA TAG TGA TGA CCT GGC CGT-3'; *ym1*: forward 5'-AGA AGG GAG TTT CAA ACC TGG G-3', reverse 5'-GTC TTG CTC ATG TGT GTA AGT GA-3'; *arginase 1*: forward 5'-CTC CAA GCC AAA GTC CTT AGA G-3', reverse 5'-AGG AGC TGT CAT TAG GGA CAT C-3'; *marc2*: forward 5'-TAC AGC TCC ACG CTA TGG ATT-3', reverse 5'-CAC TCT CCC AGT GTA GGT ACT-3'; *mgl1*: forward 5'-TGA GAA AGG CTT TAA GAA CTG GG-3', reverse 5'-GAC CAC CTG TAG TGA TGT GGG-3'; *mgl2*: forward 5'-TTA GCC AAT GTG CTT AGC TGG-3', reverse 5'-GGC CTC CAA TTC TTG AAA CCT'-3’; *lgals3*: forward 5'-TTG AAG CTG ACC ACT TCA AGG TT-3', reverse 5'- AGG TTC TTC ATC CGA TGG TTG T-3’; *TβRII*: forward 5’-GGG ATT GCC ATA GCT GTC AT-3’; reverse 5’-TGA TGG CAC AAT TGT CAC TG-3’.

### Flow cytometry and quantification of different cell populations in tissues

Data on fluorochrome-labeled monoclonal antibody-stained cells were acquired on an Accuri cytometer (Accuri Cytometers Inc). Data were analyzed using the Accuri software provided by the manufacturer. FITC-Ly6G, FITC-CD4, PE-CD8, PE-CD25, APC-CD69, APC-CD44, PE-Gal 3, PerCP-CD45, and APC-CD11b, were from BioLegend.

For quantification of different cell populations in tissues (spleen, lymph node, thymus, bone marrow and liver), tissues were harvested and processed for total live cell counts using Trypan blue and a hemacytometer. Cells were then stained with appropriate antibodies. The percentages of cell populations were calculated using Accuri software. The total cell number for each leukocyte subset was calculated by multiplying total cell counts with the percentage of that specific cell population within the total cell population.

### Efferocytosis assay

The ability of BMDM to perform phagocytosis of apoptotic cells (efferocytosis) was tested on apoptotic primary thymocytes isolated from 2-4-week old *WT* mice. After red blood cell lysis, thymocytes were cultured overnight in RPMI 1640 supplemented with 10% FBS, L-glutamine, sodium pyruvate, penicillin/streptomycin and 50 μM β-mercaptoethanol, with 6 μM CFDA. Thymocytes were induced to undergo apoptosis by treatment of 5x10^6^ cells/ml with 1 μM dexamethasone for 6 hrs, which was monitored by annexin V-FITC/propidium iodide staining and FACS. BMDM were starved for 24 hr without CSF-1 in DMEM + 15% FBS; in some experiments, polarizing cytokines were added. After a wash with cold DMEM, BMDM and thymocytes were preincubated at a ratio of 1:40 on ice. Efferocytosis was initiated by addition of prewarmed DMEM/15% FBS, followed by incubation at 37°C for different times. Controls kept on ice showed no engulfment of apoptotic thymocytes. After co-incubation, cells were washed three times in ice-cold PBS-/- and background fluorescence was quenched on ice for 15 min. with 0.25 mg/ml Trypan blue in 0.02 M ammonium acetate, 150 mM NaCl, pH 4.0. Cells were washed 3 times, fixed and stained with TRITC-phalloidin. Phagocytosis was evaluated by counting 200–300 macrophages per slide from triplicate experiments. Results for the phagocytosis index are expressed as the percentage of macrophages that had engulfed one or more thymocyte.

### Western blots

Cell lysates were resolved by SDS-PAGE and immunoblotted with anti-Arginase-1 (BD Bioscience), anti-phospho-Stat6 (Tyr641, Millipore), anti-phospho-Akt (Ser473), anti-β-catenin, and anti-iNOS antibodies (Cell Signaling). Blots were stripped and re-blotted with anti–β-actin (Sigma), anti-GAPDH (Millipore), anti-Stat6 or anti-Akt (Cell Signaling) antibodies. Band intensities were analyzed using Un-Scan-It software (Silk Scientific, Orem, UT) on the scanned images of the blots.

#### ELISA

Blood was collected from *WT* and *TβRII-/-* mice at postnatal day 18-21. After clotting of blood, serum was harvested by centrifugation at 800 g for 10 min. Serum IFNγ levels were measured using a mouse IFNγ ELISA kit (Biolegend) according to the manufacturer’s protocol.

### Statistical analysis

Data are expressed as mean ± SEM and analyzed by the unpaired Student's *t* test using Prism (GraphPad) software, unless indicated otherwise in the figure legend. *p* < 0.05 was considered to be statistically significant.

## Abbreviations

BAL, Bronchoalveolar lavage; BALF, Bronchoalveolar lavage fluid; BMDMs, Bone marrow-derived macrophages; ECM, Extracellular matrix; TGFβ, Transforming growth factor beta; TβRII, TGFβ type II receptor.

## Competing interests

The authors declare that they have no competing interests.

## Author’s contributions

DG designed and performed experiments, collected and analyzed data, and drafted the manuscript. SY designed and performed the efferocytosis and motility assays. WS and HC provided *Vav1-Cre* and *TGFβRII*^*fx/fx*^ mice and revised the manuscript. NH and JG designed and coordinated experiments, evaluated and interpreted data, and prepared the manuscript. All authors read and approved the manuscript.

## Supplementary Material

Additional file 1 Figure S1. Analysis of T-cells in thymus, spleen and lymph nodes of *WT* and *T*β*RII-/-* mice. (A) FACS of *WT* and *T*β*RII-/-* thymus for CD4- and CD8-positive cells. (B) Cell counts of double negative (DN), double positive (DP), CD4 single positive and CD8 single positive cells in thymus. (C) Percentage of CD4 + CD25+ T-cells in thymus, lymph node and spleen of *WT* and *T*β*RII-/-* mice. Solid bar, *WT*; Open bar, *T*β*RII-/-* mice. (D) *Left,* single-cell suspensions of lymph nodes were gated for lymphocytes (P1) and *right,* CD4+ and CD8+ T cells. (E) Representative histogram of CD44 expression on CD4+ T cells of *T*β*RII-/-* (red line) and control (black line) mice. (F) Percentage of CD44+ cells in spleen and lymph nodes using the strategy in (D). (G) Percentage of CD69+ cells in lymph nodes. **, p < 0.05; ***p < 0.005. Figure S2 BMDMs with or without TβRII function. (A) Gating strategy for FACS on BMDMs of the indicated genotypes using CD11b and Ly6G. *Right panel,* the expression of F4/80, a pan-macrophage surface marker. (B) BMDMs were treated with the stimuli as indicated to the right for 24 hrs. Representative phase contrast images are shown. (C) Percentage BMDMs that had engulfed at least one apoptotic thymocyte (efferocytosis) in standard medium. (D) Efferocytosis of BMDMs with or without TβRII function treated for 24 hrs with medium alone (M0); 100 ng/ml LPS and 10 ng/ml IFNγ (M1), 10 ng/ml IL-4 (M2a), or 5 ng/ml hTGFβ1. M0 compared to M1, **p = 0.003. (E) Genomic DNA was extracted from *WT* and *T*β*RII-/-* BMDMs (n = 4). Real-time PCR was used to quantify the levels of TβRII*.* The level of TβRII in one *WT* sample was set to 1 and the levels of TβRII in other samples were normalized to that of the chosen *WT* sample. Figure S3 Expression of iNOS and Arg1 in *WT* and *smad3-/-* BMDMs. Western blot analysis of Arg1 and iNOS in BMDMs of *WT* and *smad3-/-* mice. Figure S4 IFNγ levels in the blood of *WT* and *T*β*RII-/-* mice. ELISA was used to measure IFNγ levels in the serum of *WT* and *T*β*RII-/-* mice on postnatal day 18-21 (n = 4). Click here for file
