# Supplementary material for: TGFβ signaling plays a critical role in promoting alternative macrophage activation
Source: BMC Immunol. 2012 Jun 15;13:31. doi: 10.1186/1471-2172-13-31 (PMC3406960; doi:10.1186/1471-2172-13-31)
Supplement: Additional file 1 — Figure S1. Analysis of T-cells in thymus, spleen and lymph nodes of WT and TβRII-/- mice. (A) FACS of WT and TβRII-/- thymus for CD4- and CD8-positive cells. (B) Cell counts of double negative (DN), double positive (DP), CD4 single positive and CD8 single positive cells in thymus. (C) Percentage of CD4 + CD25+ T-cells in thymus, lymph node and spleen of WT and TβRII-/- mice. Solid bar, WT; Open bar, TβRII-/- mice. (D) Left, single-cell suspensions of lymph nodes were gated for lymphocytes (P1) and right, CD4+ and CD8+ T cells. (E) Representative histogram of CD44 expression on CD4+ T cells of TβRII-/- (red line) and control (black line) mice. (F) Percentage of CD44+ cells in spleen and lymph nodes using the strategy in (D). (G) Percentage of CD69+ cells in lymph nodes. **, p < 0.05; ***p < 0.005. Figure S2 BMDMs with or without TβRII function. (A) Gating strategy for FACS on BMDMs of the indicated genotypes using CD11b and Ly6G. Right panel, the expression of F4/80, a pan-macrophage surface marker. (B) BMDMs were treated with the stimuli as indicated to the right for 24 hrs. Representative phase contrast images are shown. (C) Percentage BMDMs that had engulfed at least one apoptotic thymocyte (efferocytosis) in standard medium. (D) Efferocytosis of BMDMs with or without TβRII function treated for 24 hrs with medium alone (M0); 100 ng/ml LPS and 10 ng/ml IFNγ (M1), 10 ng/ml IL-4 (M2a), or 5 ng/ml hTGFβ1. M0 compared to M1, **p = 0.003. (E) Genomic DNA was extracted from WT and TβRII-/- BMDMs (n = 4). Real-time PCR was used to quantify the levels of TβRII. The level of TβRII in one WT sample was set to 1 and the levels of TβRII in other samples were normalized to that of the chosen WT sample. Figure S3 Expression of iNOS and Arg1 in WT and smad3-/- BMDMs. Western blot analysis of Arg1 and iNOS in BMDMs of WT and smad3-/- mice. Figure S4 IFNγ levels in the blood of WT and TβRII-/- mice. ELISA was used to measure IFNγ levels in the serum of WT and TβRII-/- mice on postn [file 1471-2172-13-31-S1.pdf]

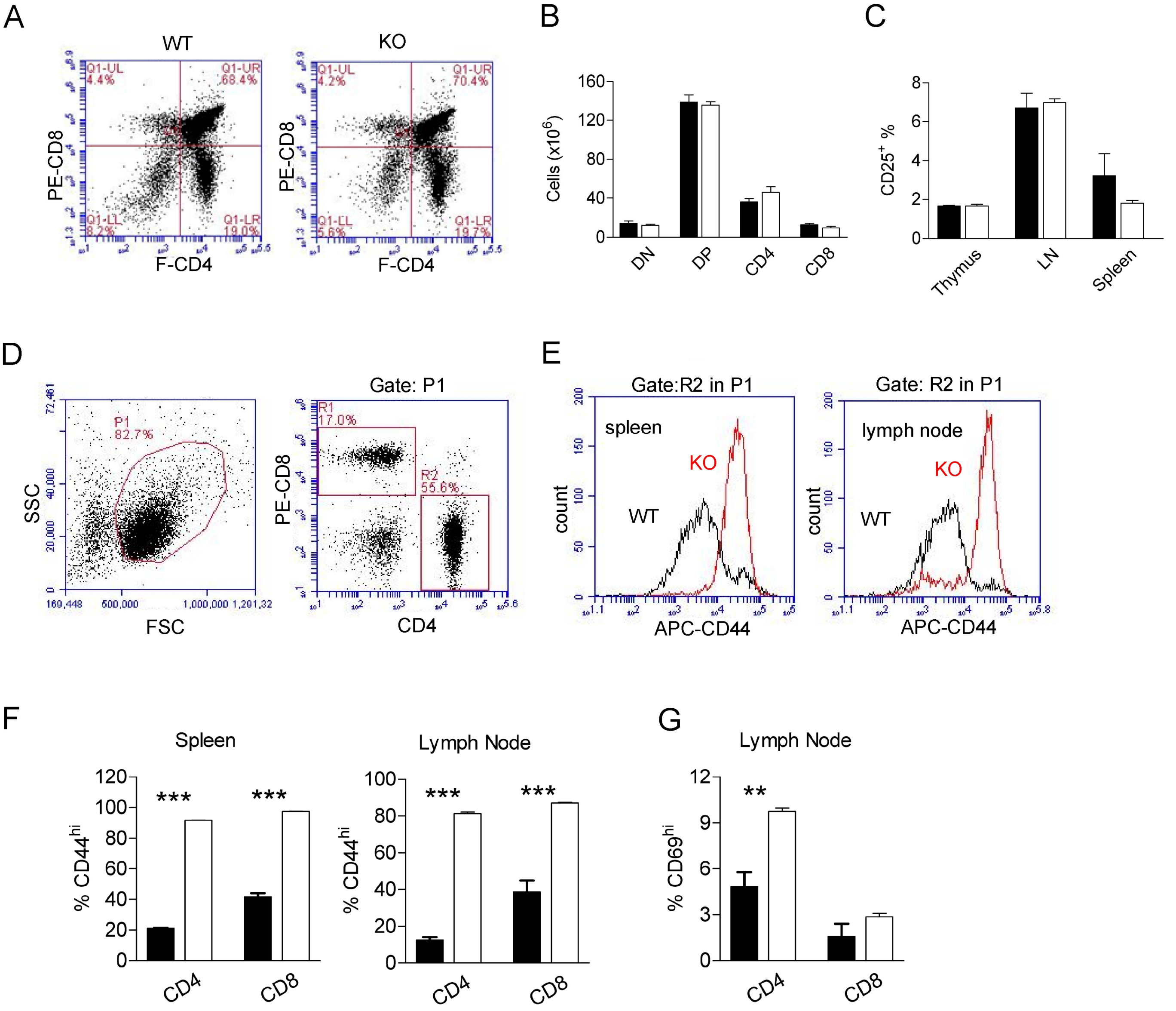

Suppl. Figure 1. **Analysis of T-cells in thymus, spleen and lymph nodes of *WT* and *TβRII-/-* mice.** (A) FACS of *WT* and *TβRII-/-* thymus for CD4- and CD8-positive cells. (B) Cell counts of double negative (DN), double positive (DP), CD4 single positive and CD8 single positive cells in thymus. (C) Percentage of $CD4^{+}CD25^{+}$ T-cells in thymus, lymph node and spleen of *WT* and *TβRII-/-* mice. Solid bar, *WT*; Open bar, *TβRII-/-* mice. (D) *Left,* single-cell suspensions of lymph nodes were gated for lymphocytes (P1) and *right,* $CD4^{+}$ and $CD8^{+}$ T cells. (E) Representative histogram of CD44 expression on $CD4^{+}$ T cells of *TβRII-/-* (red line) and control (black line) mice. (F) Percentage of $CD44^{+}$ cells in spleen and lymph nodes using the strategy in (D). (G) Percentage of $CD69^{+}$ cells in lymph nodes. **, $p<0.05$; ***$p<0.005$.

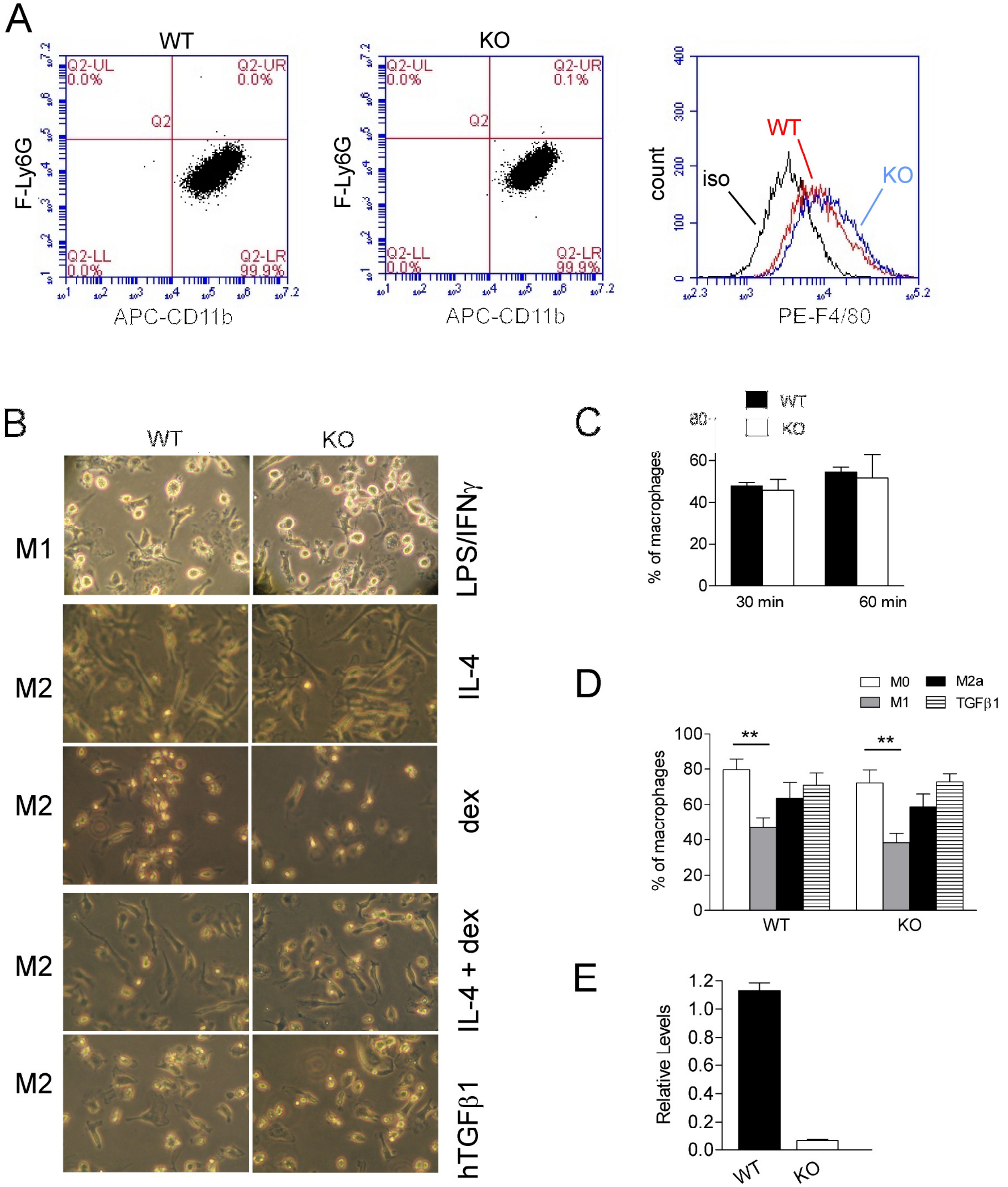

Suppl. Figure 2. BMDMs with or without TβRII function. (A) Gating strategy for FACS on BMDMs of the indicated genotypes using CD11b and Ly6G. *Right panel,* the expression of F4/80, a pan-macrophage surface marker. (B) BMDMs were treated with the stimuli as indicated to the right for 24 hrs. Representative phase contrast images are shown. (C) Percentage BMDMs that had engulfed at least one apoptotic thymocyte (efferocytosis) in standard medium. (D) Efferocytosis of BMDMs with or without TβRII function treated for 24 hrs with medium alone (M0); 100 ng/ml LPS and 10 ng/ml IFNγ (M1), 10 ng/ml IL-4 (M2a), or 5 ng/ml hTGFβ1. M0 compared to M1, **p=0.003. (E) Genomic DNA was extracted from *WT* and *TβRII-/-* BMDMs (n=4). Real-time PCR was used to quantify the levels of TβRII. The level of TβRII in one *WT* sample was set to 1 and the levels of TβRII in other samples were normalized to that of the chosen *WT* sample.

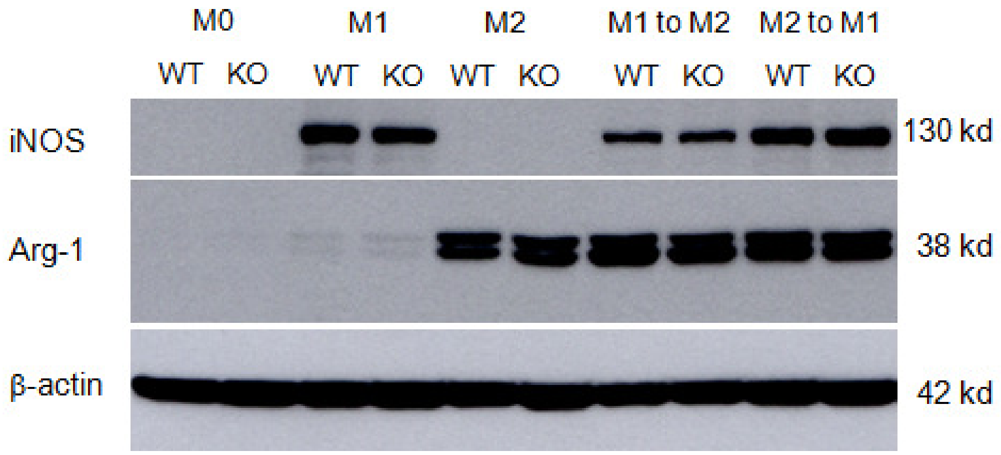

Suppl. Figure 3. Expression of iNOS and Arg1 in *WT* and *smad3-/-* BMDMs. Western blot analysis of Arg1 and iNOS in BMDMs of *WT* and *smad3-/-* mice.

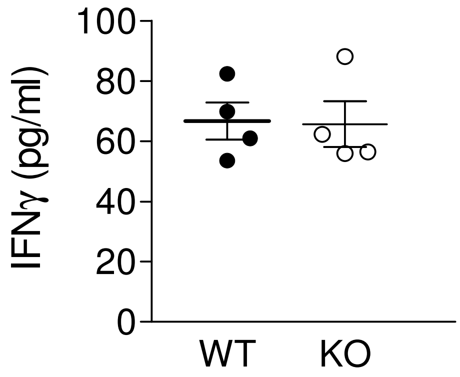

Suppl. Figure 4. IFNγ levels in the blood of *WT* and *TβRII-/-* mice. ELISA was used to measure IFNγ levels in the serum of *WT* and *TβRII-/-* mice on postnatal day 18-21 (n=4).
